# Evaluation of the efficacy of creatine chemical exchange saturation transfer imaging in assessing testicular maturity

**DOI:** 10.1002/rmb2.12507

**Published:** 2023-02-23

**Authors:** Sohei Kuribayashi, Shinichiro Fukuhara, Go Tsujimura, Takahiro Imanaka, Koichi Okada, Norichika Ueda, Kentaro Takezawa, Hiroshi Kiuchi, Shigeyoshi Saito, Yusuke Takahashi, Hidetaka Kioka, Seiya Oura, Keisuke Shimada, Masahito Ikawa, Norio Nonomura

**Affiliations:** ^1^ Department of Urology Osaka University Graduate School of Medicine Suita Japan; ^2^ Department of Medical Physics and Engineering, Division of Health Sciences Osaka University Graduate School of Medicine Suita Japan; ^3^ Department of Advanced Medical Technologies National Cerebral and Cardiovascular Research Center Suita Japan; ^4^ Department of Cardiovascular Medicine Osaka University Graduate School of Medicine Suita Japan; ^5^ Department of Molecular Pharmacology National Cerebral and Cardiovascular Center Research Institute Suita Japan; ^6^ Research Institute for Microbial Diseases Osaka University Suita Japan; ^7^ Graduate School of Pharmaceutical Sciences Osaka University Suita Japan

**Keywords:** creatine, diagnosis, magnetic resonance imaging, male infertility, testis

## Abstract

**Purpose:**

Microscopic testicular sperm extraction is the most effective treatment for NOA, but the sperm retrieval rate is low and depends on testicular maturity. However, there are limited useful tests to assess testicular maturity. Chemical exchange saturation transfer (CEST) imaging is a new magnetic resonance imaging (MRI) technique that can image the distribution of trace substances in vivo. We focused on the potential role of creatine (Cr) in testes and hypothesized that Cr‐CEST could indicate intratesticular spermatogenesis.

**Methods:**

We performed Cr‐CEST by using 7T MRI on wild‐type C57B6/J mice and several types of male infertility models such as Sertoli‐cell only (SCO) (Kit^w^/Kit^wv^), maturation arrest (MA) (Zfp541 knockout mouse and Kctd19 knockout mouse), and teratozoospermia (Tbc1d21 knockout mouse). After performing Cr‐CEST, histological analysis was performed.

**Results:**

The SCO and MA models showed decreased CEST signal intensity (*p* < 0.05), while no reduction was observed in the teratozoospermia model (*p* = 1.0). CEST signal intensity increased as the spermatogenesis stage progressed from the SCO model to the MA and teratozoospermia models. Furthermore, CEST signal intensity was reduced in 4‐week‐old wild‐type mice with immature testes (*p* < 0.05).

**Conclusions:**

This study suggests that Cr‐CEST evaluates intratesticular spermatogenesis noninvasively and provides a new therapeutic strategy for treating male infertility.

## 1 INTRODUCTION

While the global human population is on the rise,^1^ the birthrate is declining in the United States, China, and other major countries,^2^ which is becoming a problem. Infertility is a cause of declining fertility, with one in five to six couples who wish to conceive being infertile, and male infertility accounts for 50%.^3^ Semen analysis is performed as a test for male infertility, and 1%–3% have azoospermia; the absence of sperm in the semen.^4^ Azoospermia is the most severe form of male infertility, with approximately 20% of cases being obstructive azoospermia, in which the spermatic tract, including the vas deferens, is obstructed. Most of the remaining cases are nonobstructive azoospermia (NOA), in which testicular function is impaired.^4^ Patients with NOA are commonly treated with microscopic testicular sperm extraction (micro‐TESE),^5^, ^6^ which is currently the most effective sperm retrieval method. However, the sperm retrieval rate is only approximately 30% and the procedure is highly invasive, often resulting in suffering for many patients.^4^, ^7^ The low sperm retrieval rate is attributed to a lack of preoperative tests for predicting intratesticular maturity, and the development of new tests is a major unmet need.

MRI is the imaging of protons using a magnetic field, and most protons in vivo are derived from water and fat. Therefore, MRI is an imaging of water and fat; however, evaluation of molecules at low concentrations other than water and fat has been difficult so far. Chemical exchange saturation transfer (CEST) imaging is a method of estimating the concentration of target low‐concentration molecules, such as creatine, by irradiating them with specific electromagnetic waves.^8^ CEST imaging has been increasingly reported in recent years, including APT‐CEST for amide protons, and glutamine CEST (Glu‐CEST) for glutamine. APT‐CEST has already been clinically applied for brain tumor grading,^9^, ^10^ and Glu‐CEST is expected to be used for functional imaging of nerves.^11^

Testicular creatine (Cr) levels are the second highest in the body after skeletal muscle,^12^ and it is known that Cr synthase knockout mice are infertile^13^ and that Cr in the testes is decreased in patients with Klinefelter syndrome.^14^

**FIGURE 1:**
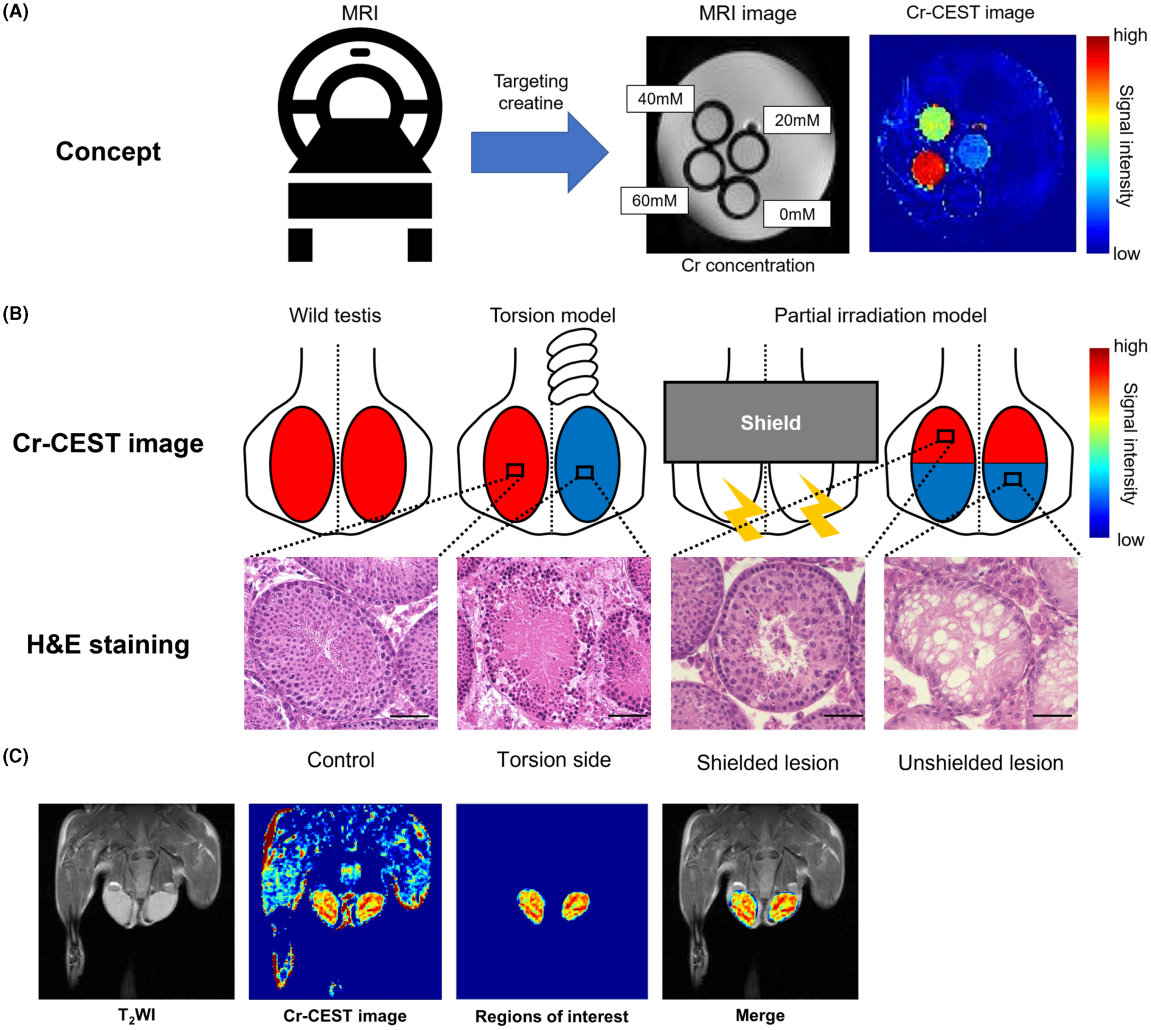
Cr‐CEST visualizes Cr distribution in testes. (A) Cr signal intensity increases with Cr concentration. (B) Schematization of our previous study.^15^, ^16^ Testicular torsion and partially irradiated models showed decreased Cr‐CEST signal intensity, consistent with pathological findings.^15^, ^16^ (C) How to create Cr‐CEST images.

Focusing on Cr in the testes, we have already successfully measured Cr in the testes using creatine CEST (Cr‐CEST) (Figure 1A).^15^, ^16^, ^17^ We have evaluated testicular injury models such as testicular ischemia,^15^ irradiation,^16^ and administration of anticancer drugs^17^ (Figure 1B). However, the testes of patients with NOA can have a wide variety of histology,^18^ including Sertoli‐cell‐only syndrome (SCO), in which no germ cells are present and only Sertoli cells are present, and maturation arrest (MA), in which germ cells are present but have not matured to spermatozoa. In addition, some NOA patients have seminiferous tubules that have only partially matured to spermatozoa.^18^ Therefore, the noninvasive evaluation of maturation status in the testes is essential because it can rule out unnecessary surgeries and accurately predict the likelihood of successful sperm retrieval.

The purpose of the present study was to develop a new noninvasive method of assessing intratesticular maturity using Cr‐CEST at the SCO, MA, and normal stages, which are clinically important to differentiate. In addition to examining intratesticular Cr at each stage of spermatogenesis, we also examined intratesticular Cr using Cr‐CEST for models in which sperm are present but sperm dysfunction exists.^19^

## 2 MATERIALS AND METHODS

### 2.1 Experimental animals

All animal procedures were approved by the Institutional Animal Care and Use Committee of Osaka University (J007559‐005). All animals were kept under standard environmental conditions (22°C, 12/12 h light/dark cycle).

Male Kit^w^/Kit^wv^, C57BL/6J, and B6D2F1 mice were supplied by Japan SLC. The Kit^w^/Kit^wv^ mouse lacks germ cells and shows only Sertoli cells in the seminiferous tubules.

Kctd19 KO mice were used as models for maturation arrest (MA) and were generated using the methodology previously described in Ref.^20^ Kctd19 KO mice exhibited typical metaphase I–arrested phenotype. In addition, ZFP541 KO mice were generated as a model in which differentiation stops early in meiosis. Tbc1d21 KO mice were used as a model of teratozoospermia and were generated using the methodology previously described in Ref.^19^

Kit^w^/Kit^wv^, Zfp541 KO, and Kctd19 KO mice underwent MRI imaging at 4 weeks of age because their testes atrophy considerably with age. Tbc1d21 KO mice were imaged at 40 weeks of age.

### 2.2 Generation of Zfp541 KO mice

**FIGURE 2:**
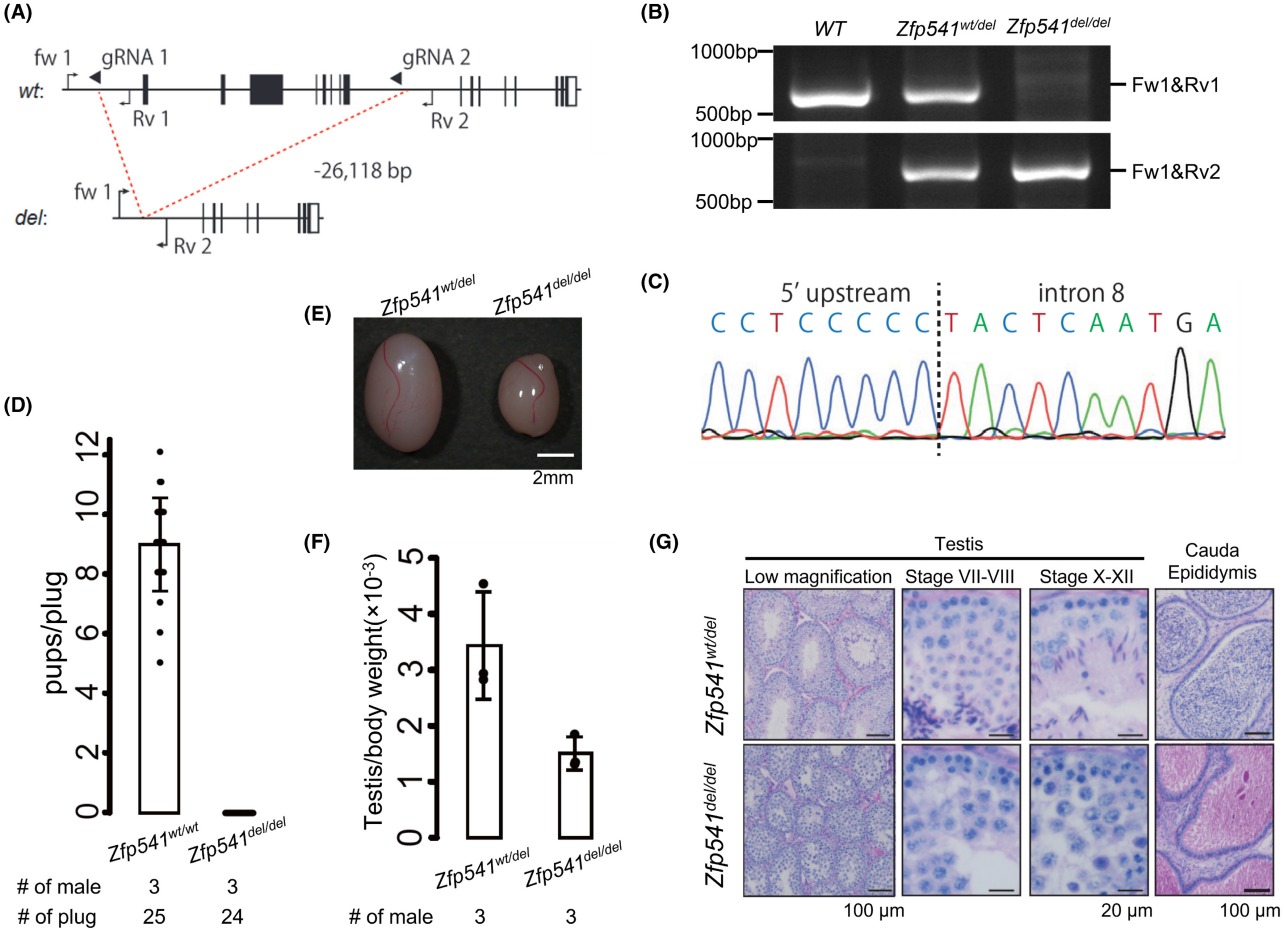
Zfp541 deficiency caused azoospermia due to meiotic defects. (A) Gene map of Zfp541. Black and white boxes indicate coding and noncoding regions, respectively. Black arrows and arrowheads indicate primers for genotyping and guide RNAs (gRNAs) for genome editing, respectively. (B) An example of genotyping PCR with two primer sets shown in (A). (C) DNA sequencing for deletion verification. (D) The result of mating tests. Pups/plug: 8.9 ± 1.6 [wt/wt]; 0 [del/del]. Error bars indicate standard deviation. (E) Testis morphology and (F) testis/bodyweight of Zfp541wt/del and Zfp541del/del adult mice. Testis/body weight: 3.4 ± 1.5 × 10–3 [wt/del], 1.5 ± 0.3 × 10–3 [del/del]. Error bars indicate standard deviation (SD). (G) Periodic acid–Schiff (PAS) staining of testicular and cauda epididymal sections (adult mice). The seminiferous epithelium cycle was determined by germ cell position and nuclear morphology.

Zfp541 mutant embryonic stem (ES) cells were generated using methods previously described^21^, ^22^ (Figure 2). EGR‐G01^23^ ES cells were transfected with two pX459 plasmids (Addgene plasmid #62988; target sequence: 5′‐CTGGTCTCAAGCTCACTAAG‐3′ and 5′‐TTATATGCAATACCCAGCAT‐3′), and colonies were cloned after transient puromycin selection. ES cells with normal karyotypes were injected into ICR mouse embryos, and chimeric blastocysts were transferred into the uteri of surrogate mothers to produce chimeric mice. Then, the obtained chimeric mice were mated with WT B6D2F1 to generate heterozygous mutant mice. A mouse colony with a 26 118 bp deletion was maintained by sibling mating. Zfp541 KO male mice exhibited recurrent DNA damage responses at the late pachytene stage, consistent with previous research.^20^ The mutant mouse line will be deposited at the Riken BioResource Center (Riken BRC) and the Center for Animal Resources and Development, Kumamoto University (CARD, Kumamoto, Japan). The genotyping primers (GeneDesign) are listed below.

Fw1: 5′‐ATTAAGAGCAACGGATGCTC‐3′
Rv1: 5′‐AGACACCCAAATCGCAGTCC‐3′
Rv2: 5′‐CTGACAGGACGGCTAGAAAG‐3′

### 2.3 MRI experiments

MR images of mice testes were acquired using a horizontal 7T scanner (PharmaScan 70/16 US; Bruker Biospin) equipped with a 30 mm inner diameter volume coil. All MR experiments on mice were performed under general anesthesia induced and maintained with 3.0% and 2.0% isoflurane, respectively. To acquire MR images, the mice were placed in a stereotaxic frame fixed at the mouth to prevent movement during acquisition. All mice were posed in the prone position, and MR images of bilateral testes were acquired in coronal slices. To prevent the testes from moving into the abdominal cavity, imaging was performed with light abdominal compression. The body temperature of the mice was maintained at 36.0 ± 0.5°C by circulating water on a heating pad and was continuously monitored using a physiological monitoring system (SA Instruments Inc.).

CEST imaging was performed to evaluate changes in the CEST effect in each testis as described in previous reports.^15^, ^16^ The pulse sequence for CEST imaging consisted of magnetization transfer with the rapid acquisition with relaxation enhancement (RARE), modified to saturate a range of frequency offsets. The sequence parameters were as follows: field of view, 32.0 × 32.0 mm^2^; slice thickness, 1 mm; repetition time (TR), 2500 ms; effective echo time (TE), 25 ms; matrix size, 196 × 196; the number of averages, 1; in‐plane resolution, 163 × 163 μm^2^; number of slices, 1; RARE factor, 8; K‐lines order, liner order. The Z‐spectra were collected using a 2000‐ms saturation pulse (pulse shape = block pulse, pulse length = 100 ms, pulse bandwidth = 12.8 Hz, number of pulses = 20, interpulse delay = 0.01 ms, Module duration = 2002 ms) at a B_1_ amplitude of 1.2 μT, with frequencies varying from −4.8 to +4.8 ppm (step, 0.3 ppm, 33 images). A point‐by‐point B_0_ correction was performed from −1.0 to +1.0 ppm (step, 0.1 ppm, 21 images) with the water saturation shift referencing (WASSR) method. The reference image (S0) was obtained without CEST saturation. A total of 55 images, including a B_0_ mapping image, were acquired in approximately 55 min. The magnetization transfer ratio (MTR) curve (or Z‐spectrum) was obtained from the CEST image series. Further, MTR asymmetry maps were reconstructed at a 1.8 ppm concentration of Cr metabolites using all 55 images. All image processing and data analyses were performed using in‐house scripts written with the MATLAB R2017b software (MathWorks). B_0_ maps were used to perform pixel‐by‐pixel B_0_ correction. To quantify the signal values on the MTR map, two regions of interest were drawn on the left and right whole testes (Figure 1C).

The CEST and the T2‐weighted images obtained by S0 were superimposed to create a figure (Figure 1C).

### 2.4 Data processing

All image processing and data analysis was performed with in‐house scripts written in MATLAB R2017b (MathWorks). S_Xppm_ is defined as the signal intensity obtained by sequence with saturation pulse at Xppm. The CEST signal intensity was evaluated using magnetization transfer ratio (MTR) asymmetry analysis, determined from the following equation:

$${\mathrm{MTR}}_{\text{asym}}\;\left(\text{Xppm}\right)=\left(S_{-\text{xppm}}-S_{\text{xppm}}\right)/\text{S0}$$

The Cr‐CEST effect was evaluated at 1.8 ppm based on our previous study.^15^, ^16^, ^17^

### 2.5 Histological examination

Testes were harvested and fixed in 10% formalin fixative. The tissues were embedded in paraffin wax, sectioned at 5 μm, stained with hematoxylin and eosin (H&E), and examined using a keyence BZ‐X700 microscope (Keyence, Co).

### 2.6 Creatine assay

Intratesticular creatine was measured by a creatine assay kit (Sigma‐Aldrich, #MAK079) according to the manufacturer's instructions. Briefly, testis was rapidly homogenized and centrifuged at 13 000 *g* for 10 min at 4°C to remove insoluble material. High concentrations of proteins may interfere with the assay and were removed with a 10 kDa MWCO spin filter (Millipore). Then, the sample was measured by colorimetric assay. Creatine concentrations were calculated against a creatine standard curve and data expressed as nanograms per microliter of supernatant.

### 2.7 Statistical analysis

All data were analyzed using JMP® 15 (SAS Institute Inc.). All data are presented as the mean ± standard error of the mean (SEM), and *p*‐values < 0.05 were considered statistically significant. Two‐tailed Student's *t*‐test for experiments with two groups and the Tukey–Kramer method for experiments including ≥3 groups were used for analysis as appropriate. *p < 0.05; **p < 0.01; ***p < 0.001; ****p < 0.0001.

## 3 RESULTS

### 3.1 Zfp541 KO mice showed male infertility

Zfp541 is a gene specifically expressed in the testes and is known to stop sperm maturation at the pachytene stage.^20^ Zfp541 KO mice showed infertility and a marked decrease in testicular weight (Figure 2D–F). PAS staining showed no round spermatocytes, suggesting that sperm maturation ceased at the pachytene stage (Figure 2G). In the cauda epididymis, no spermatozoa were observed (Figure 2G).

### 3.2 Evaluation of testicular pathology in male infertility models

In the present study, we used Kit^w^/Kit^wv^ mice as a model of SCO, the most severe type of NOA. Zfp541 KO and Kctd19 KO mice were used as models of MA, which is a less severe type of NOA than SCO. Zfp541 KO and Kctd19 KO are used as models of maturation arrest in early‐first (pachytene phase) and mid‐first meiosis (metaphase 1), respectively. Tbc1d21 KO mice were used as a model of teratozoospermia. In this model, sperm were formed in the testes; however, mitochondrial sheath dysplasia occurred, resulting in infertility.

**FIGURE 3:**
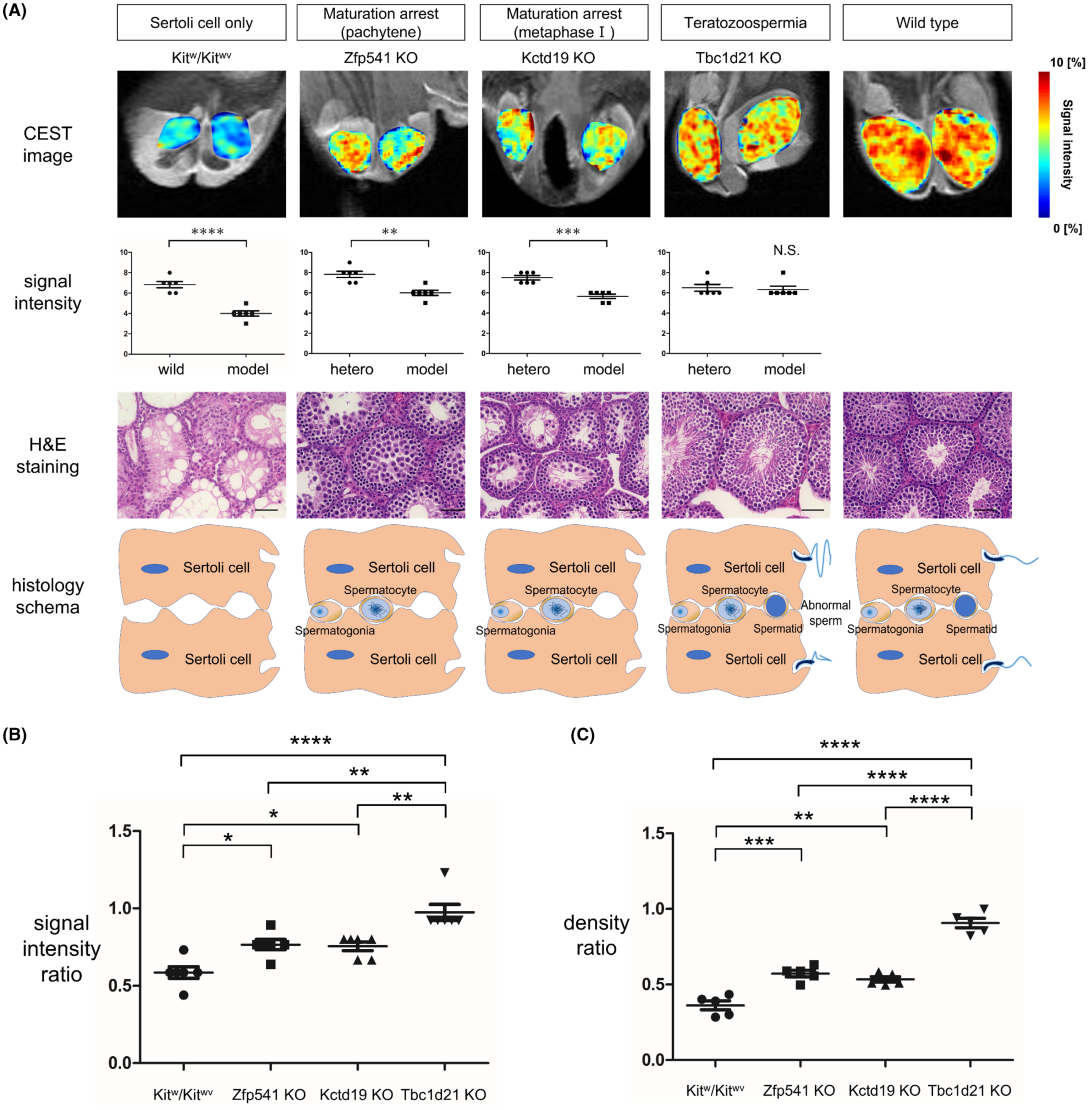
Comparison of histopathological image of male infertility models. (A) Cr‐CEST images and histopathology in the Sertoli‐cell‐only, maturation arrest, and teratozoospermia models, and comparison of signal intensity between each model and the wild type (WT) or hetero (scale bar = 50 μm). Data are expressed as the mean ± SEM (*n* = 3 mice per group). Statistical analysis was performed using the two‐tailed Student's *t*‐test. (B) Comparison of CEST signal intensity ratio for each model. The signal intensity ratio is the ratio of each group to the control. Statistical analysis was performed using the Tukey–Kramer method. (C) Comparison of cell density ratio for each model. The signal intensity ratio is the ratio of each group to the control. Statistical analysis was performed using the Tukey–Kramer method.

Kit^w^/Kit^wv^ mice had no germ cells in the seminiferous tubule and markedly smaller testes (Figure 3A). Zfp541, Kctd19 KO mice showed germ cells in the seminiferous tubule but no sperm formation (Figure 3A). The testes of Zfp541 KO and Kctd19 KO were larger than those of Kit^w^/Kit^wv^ but smaller than those of the wild type. Tbc1d21 KO mice had spermatozoa in the seminiferous tubule and no testicular atrophy (Figure 3A).

### 3.3 Cr‐CEST signal intensity decreased in NOA models

**FIGURE 4:**
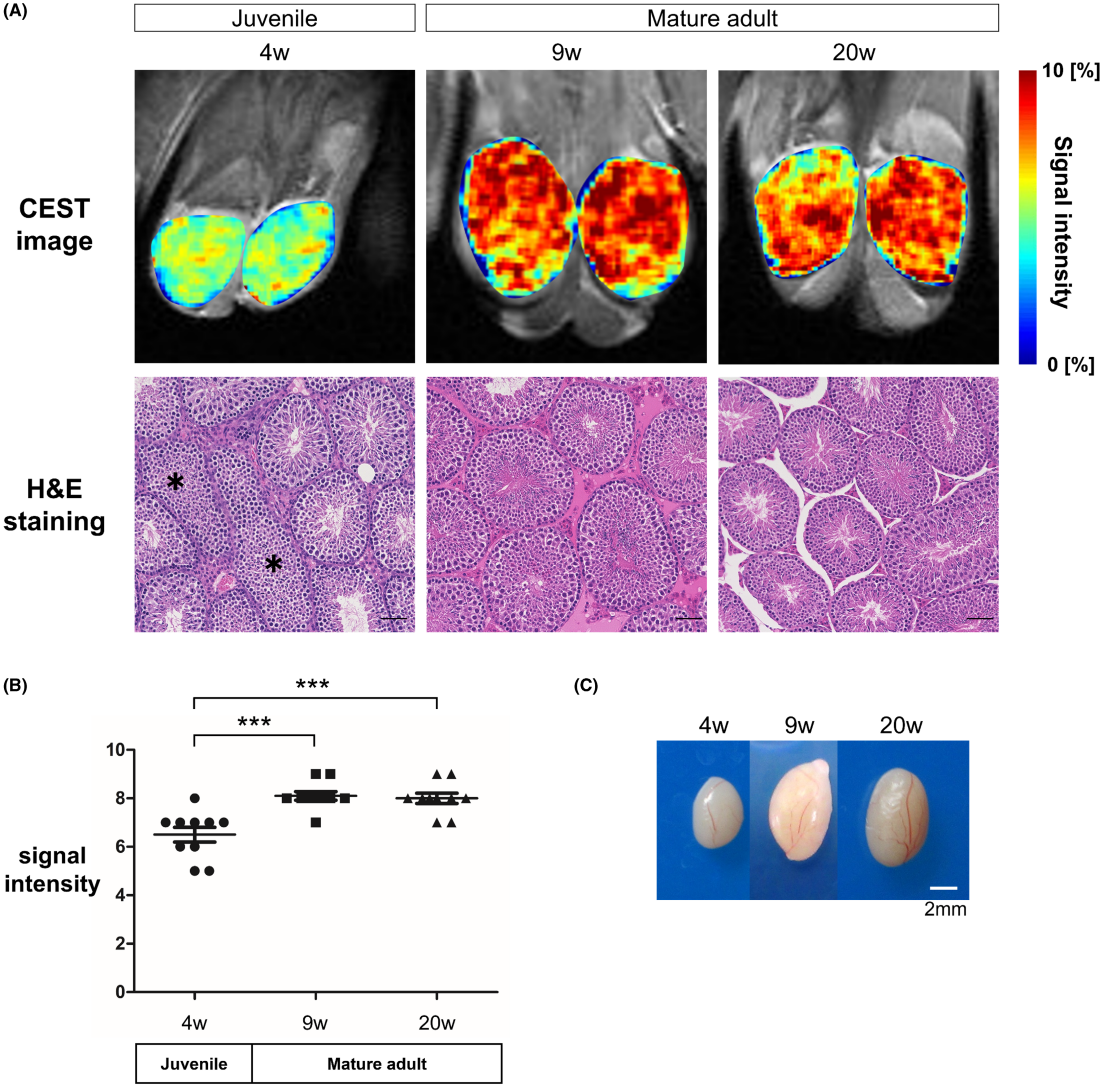
Juvenile mice showed a decrease in Cr‐CEST signal intensity. (A) Comparison of Cr‐CEST images at 4‐, 9‐, and 20‐weeks of age (Upper). Histopathological analysis of the seminiferous epithelium in 4‐week, 9‐week, and 20‐week‐old mice. 4‐week‐old mice show immature seminiferous epithelium (asterisk) (Below). (B) Change in Cr‐CEST signal intensity at each age. Statistical analysis was performed using the Tukey–Kramer method. (C) Morphological changes in the testis as age progressed from 4‐ to 20‐weeks of age.

SCO model Kit^w^/Kit^wv^ mice showed a significant decrease in CEST signal intensity compared to the WT mice (WT mice vs. Kit^w^/Kit^wv^ mice: 6.8 ± 0.31 vs. 4.0 ± 0.26, *p* < 0.0001, *n* = 3, Figure 4A).

Zfp541 and Kctd19 KO mice showed decreased CEST signal intensity compared to control mice (Control vs. Zfp541 KO mice: 7.8 ± 0.31 vs. 6.0 ± 0.26, *p* = 0.001, *n* = 3, Control vs. Kctd19 KO mice: 7.5 ± 0.22 vs. 5.7 ± 0.21, *p* = 0.0001, *n* = 3, Figure 3A). There was no significant difference between the two strains (*p* = 1.0, Figure 3A). Contrastingly, when compared to the SCO model, the signal intensity ratio was significantly increased in the MA model (Zfp541 KO mice vs. Kit^w^/Kit^wv^ mice: *p* = 0.017, Kctd19 KO mice vs. Kit^w^/Kit^wv^ mice: *p* = 0.025, Figure 3B). As a preliminary study, we compared signal intensity and intratesticular Cr levels measured by colorimetric assay in 4 weeks old Kit^w^/Kit^wv^ and WT mouse (*n* = 1). Cr concentration of the Kit^w^/Kit^wv^ mouse was also considerably lower compared to WT mouse (Figure S1).

### 3.4 Cr‐CEST signal intensity did not decrease in the teratozoospermia model

No decrease in CEST signal intensity was observed in Tbc1d21 KO mice (Control vs. Tbc1d21 KO mice: 6.5 ± 0.34 vs. 6.3 ± 0.33, *p* = 0.73, *n* = 3, Figure 3A). Furthermore, Tbc1d21 KO mice showed a significantly increased signal intensity ratio compared to the SCO and MA models (Figure 3B). Based on the histopathology image and Cr‐CEST signal, we considered that the signal intensity depends on the cell density. A comparison of cell density between Tbc1d21 mice and SCO‐ and MA‐model mice showed that cell density was significantly increased in Tbc1d21 mice (Figure 3C).

### 3.5 Cr‐CEST signal intensity increased with testicular maturity in wild‐type mice

We then examined whether Cr‐CEST signal intensity changes with maturation in the testes of WT mice, in which sexual maturation develops normally. Cr‐CEST signal intensity changes were examined by age in weeks. Testicular maturation was not complete in 4‐week‐old mice, and testes were smaller in size compared to 9‐ and 20‐week‐old mice (Figure 4C). Furthermore, hematoxylin and eosin (H&E) staining showed some seminiferous tubules with no spermatozoa (Figure 4A). The signal intensity was significantly lower in the 4‐week‐old mice than in the 9‐ and 20‐week‐old mice (4‐week‐old: 6.5 ± 0.31, 9‐week‐old: 8.1 ± 0.18, 20‐week‐old: 8.0 ± 0.21, *n* = 5, 4‐week‐old vs. 9‐week‐old: *p* = 0.0002, 4‐week‐old vs. 20‐week‐old: *p* = 0.0004, Figure 4B).

## 4 DISCUSSION

This study found that Cr‐CEST was an effective new method for assessing testicular maturity. Cr‐CEST signal intensity was reduced in SCO and MA models, similar to previous testicular damage models.^15^, ^16^ In the teratozoospermia model, Cr‐CEST signal intensity was equivalent to hetero mice with normal testicular findings. Furthermore, CEST signal intensity increased as the spermatogenesis stage progressed from the SCO model to the MA and teratozoospermia models. Similarly, Cr‐CEST signal intensity in WT mice was significantly lower at 4 weeks of age (before sperm maturation was complete) than at 9–20 weeks of age (after sperm maturation was complete). This indicated that Cr‐CEST, that is, Cr concentration in the testes, is a suitable indicator of sperm maturation in the testes.

Cr is converted to creatine phosphate (PCr) by creatine kinase, and PCr is stored as an energy‐storage substance. In muscles, PCr is used during ATP production during exercise. Therefore, in muscle, PCr is much more abundant than Cr.^12^ On the other hand, in the testes, which have the second highest total Cr content in the body after skeletal muscle, it is not present as PCr and is mostly retained as Cr.^24^ This leads us to believe that Cr may have a role other than that of an energy‐storage substance in the testis. Creatine synthase knockout mice are known to be infertile^13^; however, the role of Cr in the testis is still not clear. In recent years, an increasing number of studies have focused on Cr, and it is known that Cr is involved in T‐cell immunity,^25^ that Cr is required for oligodendrocyte survival,^26^ and that abnormalities in the Cr transporter are observed in patients with inflammatory bowel disease (IBD).^27^ In patients with inflammatory bowel disease, Cr is reduced, causing a decrease in the tight junction function of the intestinal tract, and administration of Cr has reportedly improved symptoms, attracting attention as a novel treatment.^28^ The testes also have a tight junction in the blood‐testis barrier, and disruption of the tight junction leads to infertility.^29^ We believe that if Cr is proven to be involved in this blood‐testis barrier, it could be a novel treatment for male infertility.

In this study, the number of cells in the seminiferous tubules in the NOA models is reduced when compared to the WT, while the number of cells in the seminiferous tubules is not reduced in the teratozoospermia model because the maturation of the testes is normal. Therefore, Cr concentration in the testes may reflect cell density. In the present study, Cr‐CEST signal intensity in infertility model mice was heterogeneous in the testes, and we hypothesized that there might be a correlation between the areas of high signal intensity and cell density. Therefore, we compared the histopathology and Cr‐CEST images, and found no clear correlation between the morphology and the Cr‐CEST signal intensity. This could be due to the fact that Cr‐CEST images are imaged in 1 mm slices and tissue images are sectioned in 5 μm slices, making it difficult to evaluate them in the same slice. We have already studied and reported on the spatial resolution of Cr‐CEST.^16^ We performed partial irradiation of only the lower half of the mouse testis, and compared to the nonirradiated area, the irradiated area showed a significant decrease in Cr‐CEST signal intensity, consistent with the histopathology image. In view of the high spatial resolution of Cr‐CEST, the fact that the Cr‐CEST signal intensity was heterogeneous within the testes may indicate that Cr concentrations are heterogeneous within the testes in the mouse used in this study. Although this study could not clarify the heterogeneity of Cr‐CEST signal intensity in the testis, it is conceivable that differences in Cr concentration in the seminiferous tubules may have an important role in spermatogenesis, and further studies on the role of Cr in the testis are expected in the future.

NOA can be divided into SCO (no germ cells present in the testes), and MA (germ cells are present but not yet differentiated into spermatozoa). The sperm retrieval rate of micro‐TESE for SCO is quite low, while the sperm retrieval rate for MA is increased with treatment.^30^, ^31^ The success rate of micro‐TESE will increase if SCO and MA can be distinguished in preoperative examinations. In addition, NOA patients have heterogeneous maturity within the testes, and some patients have areas of normal sperm formation.^18^ Considering that Cr‐CEST can predict testicular maturity, the use of Cr‐CEST as a preoperative navigation system could increase the sperm retrieval rate of micro‐TESE and reduce unnecessary surgery. In the future, we would like to conduct clinical trials in humans and apply them to a navigation system for micro‐TESE.

This study has several limitations. First, it was impossible to examine Cr‐CEST in mice younger than 4 weeks because they could not tolerate prolonged anesthesia. Second, in the examination of the infertility models, we performed Cr‐CEST at 4 weeks old, before maturity was complete. In the infertility models, the testes had atrophied considerably after 4 weeks old, making MRI imaging difficult; therefore, this study was conducted at 4 weeks old. Third, Cr‐CEST was not applied to humans. Cr‐CEST of mouse testes was imaged using 7T MRI; however, most MRIs used in humans are 3T. Since 7T is the most common MRI for animal experiments, it was not possible to conduct a study at 3T. Therefore, it is necessary to investigate the use of 3T MRI in the future.

In conclusion, this study showed that Cr‐CEST is a useful and novel noninvasive method for assessing maturity in the testis.

## CONFLICT OF INTEREST STATEMENT

The authors declare no conflict of interest.

## ANIMAL STUDIES

All animal procedures were approved by the Institutional Animal Care and Use Committee of Osaka University (J007559‐005).

## Supporting information


**Figure S1.** Comparison of Cr‐CEST signal intensity and testicular creatine concentration. (a) Comparison of Cr concentration of testis measured by colorimetric assay between Kit^w^/Kit^wv^ mouse and WT mouse. (b) Comparison of Cr‐CEST signal intensity between Kit^w^/Kit^wv^ mouse and WT mouse.
