# Supplementary figures and images for: Evaluation of the efficacy of creatine chemical exchange saturation transfer imaging in assessing testicular maturity
Source: Reprod Med Biol. 2023 Feb 23;22(1):e12507. doi: 10.1002/rmb2.12507 (PMC9949363; doi:10.1002/rmb2.12507)

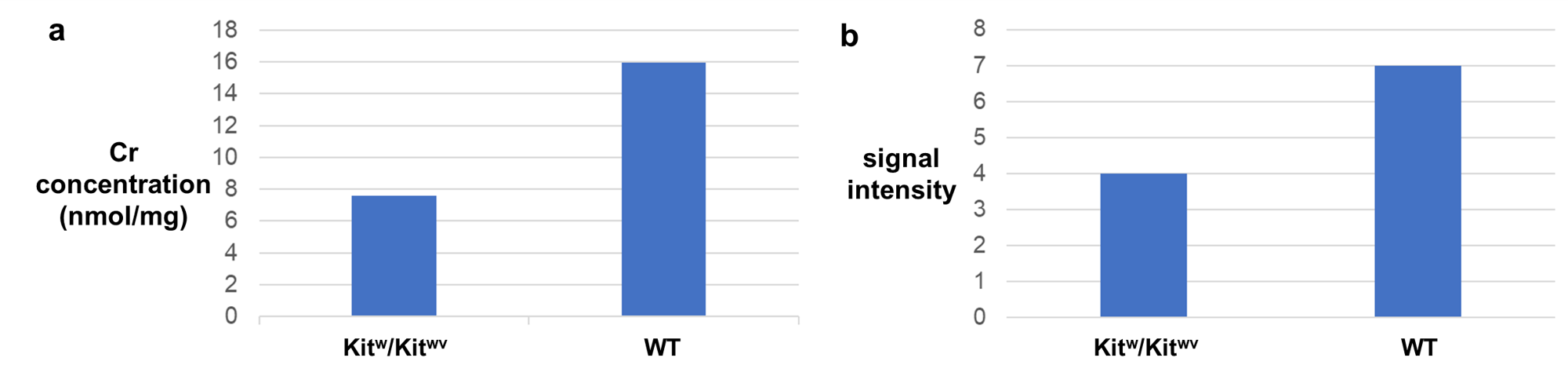

Supplement: Supplementary file 1 — Figure S1. Comparison of Cr‐CEST signal intensity and testicular creatine concentration. (a) Comparison of Cr concentration of testis measured by colorimetric assay between Kitw/Kitwv mouse and WT mouse. (b) Comparison of Cr‐CEST signal intensity between Kitw/Kitwv mouse and WT mouse. [file RMB2-22-e12507-s001.tif]
